# A retrospective study on adverse events of intravenous administration of sulfur hexafluoride microbubbles in abdominal and superficial applications in 83,778 patients

**DOI:** 10.1186/s13244-024-01632-9

**Published:** 2024-02-27

**Authors:** Di Li, Rui Zhang, Huixia Lan, Mianni Chen, Zhenli Huang, Huijuan Zhao, Shan Guo, Ming Xu, Yangyang Lei

**Affiliations:** Department of Medical Ultrasonics, Institute of Diagnostic and Interventional Ultrasound, The First Affiliated Hospital of Sun Yat-sen University, Guangzhou, China

**Keywords:** Safety, Adverse effects, Contrast media, Sulfur hexafluoride microbubbles, Ultrasonography

## Abstract

**Objectives:**

To investigate the rate of adverse events (AEs) caused by intravenous administration of sulfur hexafluoride microbubbles in abdominal and superficial applications retrospectively and to explore practical measures for prevention and treatment of them.

**Materials and methods:**

This study enrolled 83,778 contrast-enhanced ultrasound (CEUS) examinations using sulfur hexafluoride microbubbles intravenously performed during 11 years. Age, gender, and target organs of all CEUS patients were recorded. For cases of AEs, their medical history and laboratory results were also collected. The process of AEs was assessed and categorized. Besides, the management of AEs were recorded.

**Results:**

Twenty patients had sulfur hexafluoride microbubbles-related AEs. The AE rate was 0.024%. No significant difference was observed between patients with AEs and the whole group for age and sex distribution. All AEs happened in liver examinations. Among them, 7 (35%) were mild, 8 (40%) were moderate, and 5 (25%) were severe. They were categorized into 15 allergic-like reactions and 5 physiologic reactions. The manifestations of mild and moderate AEs mainly include urticaria, chills, and mild hypoxia, which could be eased by simple management. Severe cases had anaphylactic shock, generalized convulsions, and diffuse erythema with hypotension respectively. They need close monitoring and oxygen inhalation with anti-shock and anti-anaphylactic treatment. Most cases started within 30 min and recovered within 1 day.

**Conclusions:**

Intravenous administration of sulfur hexafluoride microbubbles in abdominal and superficial applications was safe with rare AEs. AEs were more likely to happen in abdominal applications than superficial ones. A well-designed emergency plan should be available for clinical use of sulfur hexafluoride microbubbles to reduce AEs and to deal with AEs properly.

**Critical relevance statement:**

Intravenous administration of sulfur hexafluoride microbubbles in abdominal and superficial applications reported few AEs and could be considered safe but severe AEs are life-threatening. We analyzed the influence factors of AEs and propose some methods for prevention and treatment of them, which can further improve the safety of sulfur hexafluoride microbubbles in clinical practice.

**Key points:**

• The AE rate of sulfur hexafluoride microbubbles in abdominal and superficial applications was 0.024%.

• Patients were more likely to have AEs in abdominal applications than superficial ones.

• Severe AEs are life-threatening and need prompt identification and treatment.

• We summarized some detailed suggestions for clinical prevention and treatment of AEs.

**Graphical Abstract:**

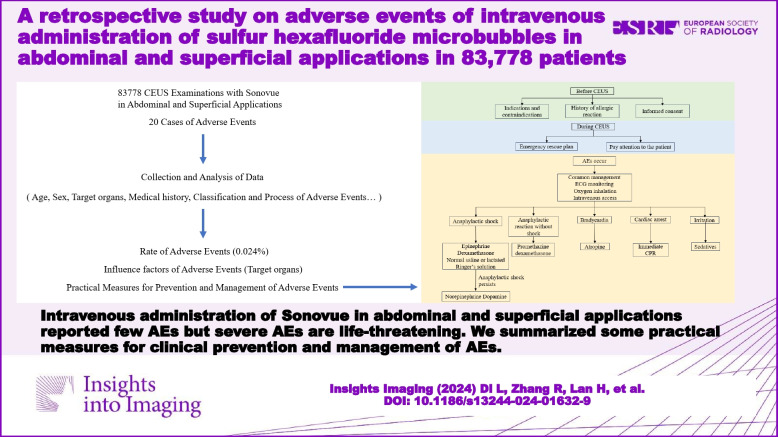

**Supplementary Information:**

The online version contains supplementary material available at 10.1186/s13244-024-01632-9.

## Introduction

Contrast-enhanced ultrasound (CEUS) is increasingly accepted in abdominal and superficial applications and serves as an important supplement to gray-scale and color doppler ultrasound in the evaluation of lesions [1–3]. The ultrasound contrast agents (UCAs) are suspensions of microbubbles that can enhance the image contrast of blood vessels and cavity organs like the bladder. The underlying mechanism is associated with two aspects. First, different acoustic impedance between the gaseous score and surrounding fluid causes the scattering of acoustic waves. Second, the ultrasonic waves act on microbubbles, generating non-linear oscillation of the bubbles and causing scattered ultrasound fields. This can be detected by CEUS mode [4].

Sulfur hexafluoride microbubbles is a second-generation UCA composed of microbubbles smaller than red blood cells with a stabilized phospholipid shell filled with the inert gas, sulfur hexafluoride. Currently, sulfur hexafluoride microbubbles have been approved to be used in imaging of heart, vessels, abdominal organs, and the urinary tract in China, Europe, and Canada (http://icus-society.org) with a low rate of adverse events (AEs) (0.020–0.125%) [5–8] compared to that of iodinated computed tomography (CT) contrast media (0.153–0.731%) and gadolinium-based contrast media (0.040–0.394%) [9–11]. It is maintained in circulation for a few minutes and then is eliminated through the lungs instead of kidneys [12, 13], without metabolism in the liver. Thus, it can be well tolerated by patients with renal insufficiency or liver dysfunction [14, 15]. However, some life-threatening AEs like cardiac arrest and anaphylactic shock that need prompt treatment were still reported in the use of UCAs [8, 16].

To our knowledge, there is no previous study that provided a simple and feasible strategy for the prevention and management of sulfur hexafluoride microbubbles-related AEs. Therefore, on the basis of the aim to describe the AE rate more accurately in a large cohort, we focused on the influence factors, manifestations, and management of AEs. By this way, we hope to summarize some practical and straightforward measures to improve the prevention and treatment effectiveness of AEs referring to the experience of our center.

## Materials and methods

The protocol of this retrospective study was approved by the Institutional Review Board of the First Affiliated Hospital of Sun Yat-Sen University. Informed consent from patients could be waived for the retrospective study, but all patients has signed informed consent to the CEUS examination using sulfur hexafluoride microbubbles as the contrast agent.

### Study participants

We carried out a retrospective study on all CEUS examinations with intravenous administration of sulfur hexafluoride microbubbles from December 2011 to March 2023, at the First Affiliated Hospital of Sun Yat-Sen University. All patients with or without adverse events soon after CEUS examinations were enrolled in this study.

### Procedures

The contrast agent used in this study, sulfur hexafluoride microbubbles, were supplied as a sterile lyophilized powder (25 mg) in a gaseous atmosphere (SF6 59 mg) in 10-mL vials. Before the administration of the contrast agent, 5 mL 0.9% sterile saline was injected into the vial which was then shaken up quickly to make the powder disperse completely into a homogeneous milky white suspension. At the beginning of CEUS examination, the suspension was aspirated into a syringe and administered intravenously through a large antecubital vein within 1 s. Another 5 mL normal saline was then injected within another 1 s to ensure all the contrast agent was administered. The doses of microbubbles suspension were 2.4 mL for examination of abdominal organs including liver, biliary system, spleen, pancreas, and kidney, 4.8 mL for breast gland, and 1.2 mL for thyroid gland in adults. For children, the dose is based on body weight, 0.03 mL/kg, no more than the dose of adults per injection [17]. After CEUS examination, all the patients were instructed to stay in the ultrasound department for 30 min. After that, our nursing staff would routinely ask the patients about possible adverse reactions and inform them that they could return to the hospital if they felt any discomfort. Then, the patients were allowed to leave the hospital.

### Data collection

Age, gender, and target organs of all patients who underwent CEUS examinations were recorded. Additionally, for cases of AEs, their history of underlying diseases and allergic reactions were also collected. We made a detailed record of the respective signs and symptoms of AEs and categorized them into allergic-like or physiologic reactions. Events were also classified into three levels of severity (mild [self-limited], moderate [not life-threatening but requiring treatment], or severe [life-threatening and requiring treatment]). The classification by type and severity was performed according to ACR (American College of Radiology) Manual On Contrast Media [18]. Besides, time from the administration of microbubbles to onset of AEs, the duration, and medical management of adverse events were recorded. Laboratory results collected included routine blood examination, liver, and renal function within 1 day after the attack of AEs.

### Statistical analysis

All statistical analyses were performed using the SPSS software (version 26.0). Continuous measurements were presented as mean (SD) if they are normally distributed or median (IQR) if they are not and categorical variables as frequencies (percentages). The Shapiro-Wilk test and Kolmogorov-Smirnov test were used to test the normality of the variables. Mann–Whitney *U* test was used to compare continuous variables, and chi-square test was used to compare categorical variables between all patients who underwent CEUS examinations and patients with AEs. All the statistical analyses were two-tailed, and a *p* value less than 0.05 was considered statistically significant. For laboratory data, we also assessed whether they were within the normal range.

## Results

In total, 83,778 CEUS examinations using sulfur hexafluoride microbubbles were performed in this hospital during approximately 11 years. The mean age of these patients was 50.77 years. Among them, 47,048 (56.16%) were male and 36,730 (43.84%) were female. Twenty patients experienced AEs after CEUS examinations, resulting in an AE rate of 0.024%. Most patients were men, with a mean age of 46.65 years (±14.46 years). There was no significant difference between patients with AEs and the whole patient group for age and sex distribution. All AEs happened during CEUS examinations of abdomen. Patients who underwent CEUS examinations of abdominal organs (liver, biliary system, spleen, pancreas and kidney) experienced AEs more often than those who underwent CEUS for superficial scans (breast and thyroid gland) (*p* = 0.002) (Table 1). For cases of AEs, the target organs included the liver (17), pancreas (2), and kidney (1). Of the 20 patients who experienced AEs, 17 (85%) had chronic diseases, including hypertension, diabetes, hepatitis, malignant tumor, and/or peripheral vascular disease. Three of the patients (15%) had a history of allergic reaction (Table 2). Allergens included transfused platelets, penicillin, and cephalosporins, respectively. For all patients who underwent CEUS, all of them were asked about the indications and contraindications of CEUS before examinations, but none of them had premedication.

**Table 1:** Demographics and target organs of patients who underwent CEUS examinations with or without adverse events

Characteristics	All patients (*n* = 83,778)	Patients with AEs (*n* = 20)	*p* value
Age (years), mean (SD)	50.77 (14.45)	46.65 (14.46)	0.309
Sex, *n* (%)			0.212
Female	36,730 (43.84%)	6 (30%)	
Male	47,048 (56.16%)	14 (70%)	
Target organs, *n* (%)			0.002
Liver/biliary system/spleen/pancreas/kidney	59,191 (70.65%)	20 (100%)	
Breast and thyroid gland	24,587 (29.35%)	0 (0%)

**Table 2:** Demographics, baseline characteristics, and target organs of AE cases of different types

Characteristics	Patients with allergic-like AEs (*n* = 15)	Patients with physiologic AEs (*n* = 5)
Age (years), mean (SD)	47.33 (14.67)	44.60 (13.60)
Sex		
Female	3 (20%)	3 (60%)
Male	12 (80%)	2 (40%)
Target organ		
Liver	13 (86.67%)	4 (80%)
Pancreas	2 (13.33%)	0 (0%)
Kidney	0 (0%)	1 (20%)
Chronic medical illness		
Hypertension	2 (13.33%)	0 (0%)
Diabetes	3 (20%)	1 (20%)
Hepatitis	10 (66.67%)	1 (20%)
Malignant tumor	12 (80%)	3 (60%)
Peripheral vascular disease	0 (0%)	1 (20%)
History of allergic reaction		
Yes	2 (13.33%)	1 (20%)
No	13 (86.67%)	4 (80%)

Thirteen cases of AEs (65%) started within 1 min after the administration of sulfur hexafluoride microbubbles. Four (20%), one (5%), and two (10%) cases happened within 1–10 min, 10–30 min, and more than 30 min after administration, respectively. The median time was 1 min (range from 6 s to 24 h). Among the 20 cases of AEs, 7 (35%) were mild in severity, 8 (40%) were moderate, and 5 (25%) were severe. They were categorized into 15 allergic-like reactions and 5 physiologic ones. The signs and symptoms of mild, moderate, and severe AEs were shown in Table 3. Patients who suffered from mild allergic-like AEs experienced limited urticaria/pruritis commonly (*n* = 3). For patients with mild physiologic AEs, the most common manifestations were transient flushing/warmth/chills (*n* = 4). Two patients also complained of chest pain and headache, respectively. For patients with moderate allergic-like AEs, the most common manifestations were diffuse erythema with stable vital signs (*n* = 4), followed by diffuse urticaria/pruritis (*n* = 3) and wheezing/bronchospasm, mild or no hypoxia (*n* = 1). Apart from these symptoms, 1 patient also complained of itchy nose and change of smell. For patients with severe allergic-like AEs, the most common manifestations were anaphylactic shock (hypotension + tachycardia) (*n* = 3), followed by diffuse erythema with hypotension (*n* = 1). The patient with severe physiologic AEs suffered from an attack of generalized convulsions. Description of the onset and progression of the 5 severe AEs was summarized in Table 4.

**Table 3:** Signs and symptoms of mild, moderate, and severe adverse events categorized according to American College of Radiology Manual On Contrast Media

Classifications of adverse events	Number
Mild	
Allergic-like	
Limited urticaria/pruritis	3
Cutaneous Edema	0
Limited “itchy”/“scratchy” throat	0
Nasal congestion	0
Sneezing/conjunctivitis/rhinorrhea	0
Physiologic	
Limited nausea/vomiting limited	0
Transient flushing/warmth/chills	4
Headache/dizziness/anxiety/altered taste	0
Mild hypertension	0
Vasovagal reaction that resolves spontaneously	0
Moderate	
Allergic-like	
Diffuse urticaria/pruritis	3
Diffuse erythema, stable vital signs	4
Facial edema without dyspnea	0
Throat tightness or hoarseness without dyspnea	0
Wheezing/bronchospasm, mild or no hypoxia	1
Physiologic	
Protracted nausea/vomiting	0
Hypertensive urgency	0
Isolated chest pain	0
Vasovagal reaction that requires and is responsive to treatment	0
Severe	
Allergic-like	
Diffuse edema, or facial edema with dyspnea	0
Diffuse erythema with hypotension	1
Laryngeal edema with stridor and/or hypoxia	0
Wheezing/bronchospasm, significant hypoxia	0
Anaphylactic shock (hypotension + tachycardia)	3
Physiologic	
Vasovagal reaction resistant to treatment	0
Arrhythmia	0
Convulsions, seizures	1
Hypertensive emergency	0

**Table 4:** The detailed process of the 5 cases of severe adverse events

Case	Age, year/sex	Target organ	Onset time, duration	Manifestations	Vital signs	Treatments	Subsequent hospitalization department
1	51/male	Liver	2 min, 60 min	Hot and numbness of the body ↓ Chest tightness, tachypnea and sweating ↓ Numbness relieved, abdominal discomfort and threw up the food in the stomach ↓ Disappeared	BP 94/56, HR 100, SpO_2_ 95% ↓ BP 80/60, HR 110, SpO_2_ 90% ↓ BP 60–70/40–50, HR 180–200, SpO_2_ 70–80%, RR 26–30 ↓ Normal	High-flow oxygen inhalation ↓ IV dexamethasone(5 mg) + IH epinephrine (1 mg) ↓ IV NS (500 mL) + IV dexamethasone (5 mg) and dopamine (20 mg) + IV drip of dopamine (40 mg) ↓ IV drip of NS (250 mL)	General ward
2	61/female	Liver	10 min, 15 min	Generalized convulsions, unresponsive ↓ Regained consciousness	Unmeasurable BP and SpO_2_, HR 40, RR 50 ↓ BP 110/60, HR 120, SpO_2_ 98%, RR 25	High-flow oxygen inhalation + IH epinephrine (1 mg) + IV atropine (0.5 mg) and dexamethasone (10 mg) + IV drip of norepinephrine (12 mg) and glucose (50 mg)	General ward
3	56/female	Liver	40 s, 24 h	Hot and diffuse erythema ↓ Lost consciousness, diffuse cyanosis ↓ Regained consciousness and responsiveness	BP 90/58, SpO_2_ 80% ↓ HR 0, SpO_2_ 70% ↓ BP 110/80, HR 90–100, SpO_2_ 98% ↓ BP 88/53, HR 114^a^	High-flow oxygen inhalation + IV dexamethasone (10 mg) ↓ CPR + IV epinephrine (1 mg) + IM promethazine (25 mg) + rapid IV drip of 5% sodium bicarbonate (125 mL) ↓ Low-dose IV norepinephrine + continuous rehydration^a^	Intensive care unit
4	38/male	Liver	30 s, 24 h	Altered taste, numbness, dyspnea with cyanosis of limbs, fecal incontinence, dysphoria ↓ Chest tightness, diffuse numbness, sweating, dysphoria ↓ Loss of sight (5 min) ↓ Regained consciousness and responsiveness	BP 80/-^b^, HR 80, SpO_2_ 66–86% ↓ BP 129/79, SpO_2_ 75–99% ↓ Normal (with norepinephrine)	High-flow oxygen inhalation + IV dexamethasone (10 mg), epinephrine (0.3 mg) and lactated Ringer’s solution (500 mL) + IV epinephrine (1 mg) ↓ IM promethazine (50 mg) ↓ Infusion pump of norepinephrine (10 mg) and NS (50 mL) ↓ IV dexmedetomidine and norepinephrine^a^	Intensive care unit
5	8/male	Liver	1 min, 48 h	Unresponsive, cyanosis of mouth and skin ↓ Urticaria and pruritis of face and lower limbs, cyanosis^a^ ↓ Disappeared^a^	BP 80/60, HR 90, SpO_2_ 80% ↓ BP 108/68, HR 122, SpO_2_ 98%, RR 32 ↓ BP 107/60, HR 145, SpO_2_ 80%^a^	High-flow oxygen inhalation + IV and IM epinephrine (0.2 mg) + rapid infusion of lactated Ringer’s solution ↓ IV dexamethasone and IM chlorphenamine maleate^a^	General ward

*BP* blood pressure, *HR* heart rate, *RR* respiratory rate, *IV* intravenous, *IH* subcutaneous, *NS* normal saline, *CPR* cardiopulmonary resuscitation, *IM* intramuscular

^a^These changes of condition and treatments happened in intensive care unit or general ward

^b^The diastolic pressure was undetectable

After the attack of AEs, increased leucocytes were observed in 11 (55%) patients. Platelets were above the normal range in 4 (20%) patients and below the normal range in 3 (15%) patients. 6 (30%) patients had hemoglobin below the normal range. Albumin decreased in 9 (45%) patients, and total bilirubin increased in 2 (10%) patients. Different degrees of abnormal liver function could be observed in 14 (70%) patients, with elevated alanine aminotransferase (ALT) or aspartate aminotransferase (AST). 2 (10%) patients had different degrees of renal function abnormality, with blood urea nitrogen or serum creatinine above the normal range (Table S1).

For moderate allergic-like AEs, the standard treatment was intravenous dexamethasone and epinephrine. For one patient, intramuscular promethazine was also added. Apart from these, some symptomatic treatments were also effective, including oxygen inhalation when patients complained of dyspnea and keeping warm when patient had a chill. The detailed processes of 5 severe AEs were introduced in Table 4. Most patients (14/20, 70%) recovered within 1 day. 4 (20%) patients recovered on the second day, and 2 (10%) patients recovered on the third day.

## Discussion

Our study enrolled 83,778 CEUS examinations using sulfur hexafluoride microbubbles that were performed in our hospital over approximately 11 years. The results of the study could provide valuable experience for the prevention and treatment of AEs of sulfur hexafluoride microbubbles. It has been proven that the administration of UCAs in CEUS examination is safe [19–21]. Sulfur hexafluoride microbubbles, a commonly used UCA, was the focus of our study. The adverse events of sulfur hexafluoride microbubbles were usually associated with allergic-like reaction or nonspecific physiologic reaction. The reported AEs included rash, edema, sweating, nausea and vomiting, nasal bleeding, back pain, headache, dizziness, dyspnea, anaphylactic shock (hypotension + tachycardia), and cardiac arrest. Most of them were self-limited or resolved quickly after simple treatment. However, active management was necessary for rare severe AEs. The study was to investigate the rate of sulfur hexafluoride microbubbles-related AEs of different severity and classification in a large patient cohort. Meanwhile, through detailed review of the identification and management of AEs, especially severe ones, we aimed to provide some simple and effective measures to improve the safety of sulfur hexafluoride microbubbles, which was seldom presented in previous studies.

The overall incidence of AEs was 0.024% and relatively low compared to the previous data. No significant difference between patients with AEs and the whole was observed in age and sex aspects. But we found that all the AEs happened following examination of abdominal organs, especially the liver. No AEs were observed in examinations of superficial organs including thyroid and breast gland. This is a new finding of our study compared to previous studies. Actually, the doses of microbubbles suspension used in breast examinations were higher (4.8 mL). Thus, we speculated that the likelihood of AEs might be related to the existence of prior liver disease and not related to the dosage. Besides, deviations from normal range of laboratory results might result from prior diseases as well, especially decreased albumin and elevated liver enzymes from liver disease. As for the relationship between laboratory results and AEs, there might be 3 explanations. (1) They have no relationship because the results were not compared with those without AEs. (2) The patients with specific laboratory results and underlying diseases were more likely to have AEs after CEUS. (3) The changes of laboratory results were the effect of AEs. To draw reliable conclusions about that, more detailed research with baseline laboratory data and comparison data is needed.

We summed up that a series of measures might be helpful to keep a low rate of AEs. Before CEUS examination, the indications and contraindications should be clearly mastered, which could be found in the official package insert of the drug. In addition, patients should be asked about any history of allergic reaction, especially those about the use of sulfur hexafluoride microbubbles and its component, polyethylene glycol [22]. Risks and benefits should be made clear to the patients, and they need to sign informed consent to the CEUS examination. The above measures should be strictly enforced for each CEUS examination.

It was observed that most (90%) of the AEs started within 30 min after the administration of contrast agent. Among the 20 cases of AEs in our study, most were mild or moderate with symptoms including limited or diffuse urticaria, pruritis or erythema, chills, mild hypoxia, itchy nose, change of smell, chest pain, and headache. The patients were usually told to relax and were given routine treatments including dexamethasone, epinephrine, promethazine, and/or oxygen inhalation. They did not require hospitalization. For the 5 severe cases of AEs, most suffered from an anaphylactic shock. The other 2 cases had an attack of generalized convulsions and diffuse erythema with hypotension respectively. The common management includes immediate ECG monitoring, oxygen inhalation, and intravenous access establishment. After that, epinephrine, dexamethasone, and rapid infusion of normal saline or lactated Ringer’s solution were suggested for anaphylactic shock [23]. If the vital signs remained poor, norepinephrine or dopamine could be taken into consideration. In addition, promethazine or dexamethasone could also be used for anti-anaphylactic treatment. If the patient suffered from bradycardia, atropine could be used to raise the heart rate. If the patient suffered from a cardiac arrest, CPR should be given immediately. If the patient was irritable, some sedatives could be administered, such as dexmedetomidine. After the rescue, the 5 patients were transferred to the ward or intensive care unit for close observation. Most patients recovered within 1 day.

**Fig. 1:**
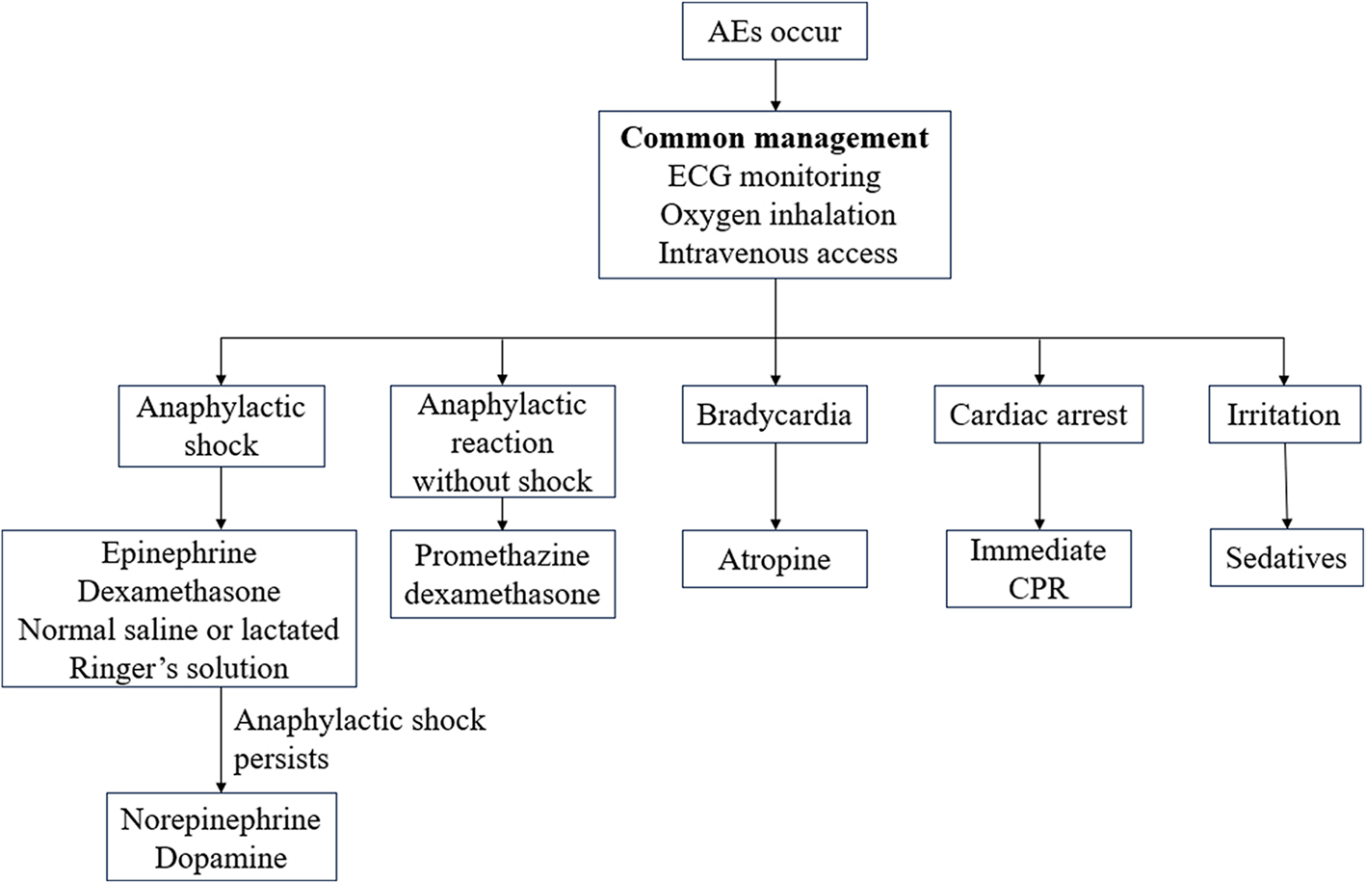
Summary of a series of solutions to deal with different situations

We learned some experience from the 20 cases of AEs. First, an emergency rescue plan for contrast agent related adverse events should be well designed. A properly trained team, common medication and equipment must be available during the CEUS examination. Second, when the doctors carry out the examination, we should pay attention to the patient’s state of consciousness, appearance of their skin, breathing, and any other discomfort. Third, after the CEUS examination, the patients should be asked to stay in the department for at least 30 min for close monitoring of any potential adverse events. Fourth, if suspected AEs occur, we summarized a series of solutions to deal with different situations (Fig. 1). Fifth, patients who suffered from mild or moderate AEs could return home if the symptoms disappeared after treatment. Patients with severe AEs required hospitalization in intensive care unit or general ward for at least 1 day. If the symptoms were persistent, the time of hospitalization would be prolonged. Before they left the hospital, all patients with AEs should be told not to have CEUS examinations again. Finally, the clinical manifestations, treatment, and prognosis of each case should be recorded in detail and followed up.

There are some limitations in our study. First, the study only enrolled cases in single center. Information from more centers can provide more universal experience. Second, this is a retrospective study with its inherent limitations. (1) The past data may introduce information bias about the quality and accuracy of the records. The data of the patients without AEs was insufficient for more comparison with cases of AEs. The lack of baseline laboratory results also affected the analysis of relationships between AEs and blood test results. (2) It is possible that some patients experienced mild AEs but did not contact us. Although these AEs were very mild and probably did not affect life, this may cause selection bias and a relatively high proportion of severe cases in all AEs. (3) It is difficult to identify and control the confounding variables that existed during our study period. This is challenging for the interpretation of the data. Third, our study only included the CEUS examinations of abdominal and superficial organs. CEUS examinations in the cardiovascular and reproductive systems have been increasing. The related AEs need further study. Fourth, we did not analyze the specific mechanism underlying AEs. The American Society of Echocardiography proposed that the AEs to US-based contrast agents may be associated with polyethylene glycol which is incorporated in the shell of Definity and Luminity and is also the excipient of Lumason and Sonovue (trade name of sulfur hexafluoride microbubbles). The possible mechanism could include 2 aspects: complement(C’) activation-related pseudoallergy (CARPA) reactions and IgE-mediated type I hypersensitivity reactions [22]. However, for the exact mechanism, further research is still needed.

In summary, our study reported a low rate of AEs caused by intravenous administration of sulfur hexafluoride microbubbles in CEUS examination of abdominal and superficial organs. This result further confirmed the safety of sulfur hexafluoride microbubbles on the basis of previous studies and found AEs were more likely to happen following abdominal applications than superficial ones. By analyzing AE cases, we summarized some experiences on clinical prevention and treatment of AEs. Before CEUS examination, indications and contraindications should be verified and allergic history should be taken in detail. A comprehensive emergency plan, a well-trained team, prepared medication and facilities would help treat the AEs properly and promptly, thus improving the prognosis of the patients in clinical practice.

### Supplementary Information

**Additional file 1:** **Table S1.** Laboratory results of patients with AEs.

## Data Availability

The raw data supporting the conclusions of this article will be available from all the authors, without undue reservation.

## Abbreviations

ACR: American College of Radiology
AE: Adverse event
ALT: Alanine aminotransferase
AST: Aspartate aminotransferase
BP: Blood pressure
CARPA: Complement(C’) activation-related pseudoallergy
CEUS: Contrast-enhanced ultrasound
CPR: Cardiopulmonary resuscitation
CT: Computed tomography
ECG: Electrocardiograph
HR: Heart rate
IH: Subcutaneous
IM: Intramuscular
IV: Intravenous
NS: Normal saline
RR: Respiratory rate
UCA: Ultrasound contrast agent
