# Supplementary material for: A retrospective study on adverse events of intravenous administration of sulfur hexafluoride microbubbles in abdominal and superficial applications in 83,778 patients
Source: Insights Imaging. 2024 Feb 27;15:65. doi: 10.1186/s13244-024-01632-9 (PMC10899544; doi:10.1186/s13244-024-01632-9)
Supplement: Supplementary file 1 — Additional file 1: Table S1. Laboratory results of patients with AEs. [file 13244_2024_1632_MOESM1_ESM.pdf]

A Retrospective Study on Adverse Events of Intravenous Administration of Sonovue in Abdominal and Superficial Applications in 83778 Patients

ELECTRONIC SUPPLEMENTARY MATERIAL

TableS1 Laboratory results of patients with AEs

	Patients (n=20)
**Blood routine**	
Leucocytes ($\times 10^9$per L; normal range 4.0–10.0)	10.9(4.6)
Increased	11(55%)
Platelets ($\times 10^9$per L; normal range 100.0–300.0)	240.3(124.5)
Increased	4(20%)
Decreased	3(15%)
Hemoglobin (g/L; normal range 115.0–175.0)	124.2(25.9)
Decreased	6(30%)
**Blood biochemistry**	
Albumin (g/L; normal range 35.0–50.0)	35.5(7.5)
Decreased	9(45%)
Alanine aminotransferase (U/L; normal range 1.0–40.0)	39.2(18.4)
Increased	6(30%)
Aspartate aminotransferase (U/L; normal range 1.0–37.0)	43.5(31.5-48.3)
Increased	13(65%)
Total bilirubin (μmol/L; normal range 3.0–22.0)	13.9(11.6-16.8)
Increased	2(10%)
Blood urea nitrogen (mmol/L; normal range 2.9–8.6)	4.5(3.7-5.6)
Increased	2(10%)
Decreased	1(5%)
Serum creatinine (μmol/L; normal range 53.0–115.0)	63.0(60.5-76.5)
Increased	1(5%)
Decreased	3(15%)
